# Supplementary material for: Evaluation of the clinical performance of a magnetic force-assisted electrochemical immunoassay for the detection of SARS-CoV-2 antigens
Source: PLoS One. 2021 Oct 7;16(10):e0258394. doi: 10.1371/journal.pone.0258394 (PMC8496795; doi:10.1371/journal.pone.0258394)
Supplement: Attachment — Submitted filename: Response_to_Reviewers.docx [file pone.0258394.s001.docx]

**Response to Reviewers**

**Comments to the Author**

**Reviewer #1:** The Authors evaluated the MARK-B COVID-19 Ag test (MARK-B, BBB, Sungnam, Republic of Korea) that is a portable point-of-care device based on the use of a magnetic force-assisted electrochemical immunoassay (MESIA) designed to detect SARS-CoV-2 antigens. Notably, this newly developed immunoassay, MESIA, reportedly suitable for measuring low concentrations of proteins for cancer detection and with the potential to detect any other biomarkers.

The evaluation was done against RT PCR as the gold standard for SARS COV-2 detection and another immunochromatographic manual antigen assay for SARS CoV-2, showing a significant higher correlation with RT PCR and a significant higher sensitivity over the standard antigen assay. Overall, this data confirms that MESIA may provide enhanced sensitivity for point-of-care devices that involve the detection of SARS-CoV-2.

The study is well conducted, each section is well written and developed, statistical measures correctly applied. There is one general limitation in this study:

The MARK-B test has not been CE/FDA-cleared or approved so far for SARS CoV-2, but these validations are pending. Therefore, this is a clear limitation of the study conducted by the Authors, in terms of extension and commercialization of this device. The Authors should discuss this issue and clarify if there are any on-going news on this important issue which is essential for the scientific community. Moreover, the test is not cited by FIND as the date of today July 15 among the 653 antigen immunoassays for SARS CoV-2 enlisted in the web site https://www.finddx.org/covid-19/pipeline/?section=immunoassays#diag
Is this just for Asia? The Authors must clarify it.

**Answer :** The MARK-B™ COVID-19 Ag has been approved by CE and Ministry of Food and Drug Safety of the Korean government for the diagnosis of SARS-CoV-2. The same test with another brand name (Sampinute COVID-19 Antigen MIA) has also been authorized for emergency use by the FDA. The MARK-B™ COVID-19 Ag has been sold in Italy, Colombia, Argentina, Ghana, and many other countries in the world based on the authorization and registration by the local regulatory authorities.

Appendix A. CE_EAR Certificate (ID032512).pdf

Appendix B. FDA_EUA Letter.pdf

Appendix C. MFDS_Export Approval Letter.pdf

Appendix D. MFDS_Approval Letter.pdf

Minor issues

Material and Methods:

The semi-quantitative/quantitation system used by MESIA for their electric signal should be specified by the Authors as they use it in the Figures, as well as the nature of the cut-off value and the type of antigen detected by this system.

**Answer :** The MARK-B™ COVID-19 Ag is used for the detection of from SARS-CoV-2 nucleocapsid protein with the MARK-B™ 1 analyzer, which is a fully-integrated immunoassay system based on the magnetic field and the electrochemical measurement. The MARK-B™ 1 analyzer is composed of a mobile device with touch screen, a battery, cooling fans, step motors, magnets, and modules for signal processing and wireless communication as shown below:

Once the sample is loaded into the test cartridge, all the processes for MESIA are automatically conducted by the MARK-B™ 1 analyzer. The test reveals the presence (positive) or absence (negative) of SARS-CoV-2 nucleocapsid proteins by measuring the electrical current and comparing it to a lot-specific cut-off value. The preset cut-off value is determined for each lot once they’re manufactured based on the mean and the standard deviation of the electrical current from the negative (blank) samples. The QR code on each cartridge contains the information and the analyzer can recognize the cut-off of each cartridge when the QR code is scanned. If the measured current from a specimen is higher than the cut-off, the analyzer will show ‘positive’ on the screen. If the current is equal to or lower than the cut-off, the analyzer will show ‘negative.’

We described this in revised manuscript (line 84-103)

**Reviewer #2:** This paper describes the evaluation of a magnetic force-assisted eletrochemical immunoassay -based test compared to other commercial rapid Ag tests by application to clinical samples. Authors have successfully compared the results provided by this device with those from other methodologies applying them to a large number of samples. In my opinion, the information offered in the article is very interesting, the electrochemical sensor has demonstrated a good behavior, but the experimental work and the new data in the article are not relevant from the point of view of scientific research. I consider that the paper should not be published in its current state in PLOs One.

**Answer :**

This is the first study describing the qualitative/quantitative detection of SARS-Cov-2 Ag using the MESIA method, and the first to conduct a clinical evaluation using MARK-B. The MESIA method can exhibit higher sensitivity than conventional rapid antigen detection methods. Although the current Ag test has a lot of usefulness, its use is limited due to its relatively low sensitivity. Although RT-PCR has high sensitivity, it is also limited in use. Our research aims to provide information that can overcome the shortcomings of the Ag test. In addition, in medical sites, the Ct value of RT-PCR changes are observed in the patient's condition. I think the information on MARK-B that we studied this time will be helpful in a situation where RT-PCR cannot be operated.

**Reviewer #3:** This is a clear well-written paper describing the performance of a new Ag test for diagnosis of COVID-19 infection. As an independent of assessment of the test, the publication provides important data to users who may be considering use of the test. I recommend publication after revision to address some minor weaknesses in the paper:

1. The main weakness is associated with the comparison to an existing commercial Ag test and the interpretation of the comparison

a. In the abstract and main text their are locations where the authors describe their paper as comparing their test to commercial antigen tests. These statements should be clarified to indicate that they compared to a (as in one) antigen test, and isn't necessarily reflective of how the test would perform to the range of available tests.

b. Some discussion is warranted regarding the criteria for the selection of the comparator test, and the relative performance of this test. Relative to the many Ag tests that are now available commercially, does the published data for the SDQ test place it at the top, middle or bottom of the range for clinical sensitivity and accuracy?

c. It would be helpful to provide some testing with a reference sample (for example, one of the available viral culture reference samples or a sample of recombinant nucleocapsid protein) that could allow direct comparison of the analytical performance of the antigen assays to each other (and potentially other commercial tests) and determination of whether the clinical performance is a direct reflection of the analytical performance).

**Answer :**

**a. b :** The Standard Q COVID-19 Ag Test is manufactured by SD Biosensor, Inc. and is similar to the Roche’s SARS-CoV-2 Rapid Antigen Test, which has been widely sold in markets accepting the CE mark [<https://www.roche.com/media/releases/med-cor-2020-09-01b.htm>].

References showing the performance of Standard Q COVID-19 Ag Test:
<https://pubmed.ncbi.nlm.nih.gov/33724916/>
<https://jkms.org/DOIx.php?id=10.3346/jkms.2021.36.e101>
<https://search.bvsalud.org/global-literature-on-novel-coronavirus-2019-ncov/resource/en/covidwho-1094070>
<https://www.sciencedirect.com/science/article/pii/S120197122032275X>

**c:** We conducted an additional experimental using reference viral culture samples. That was conducted to compare the LOD of MARK-B and SDQ by diluting viral culture samples in a laboratory that satisfies BSL-3. For this reason, RT-PCR which has the same setting as ours, could not be conducted.

We described this in revised manuscript (line 129-138, 256-259)

2. The volume of UTM used to extract the sample should be provided and, if applicable, a discussion of how the volume of UTM may differ from the volume of extraction buffer used in the standard swab protocols for the MESIA and SDQ tests, and how that may affect performance.

**Answer :** The manufacturer recommends using 400 μL of UTM or a direct swab to extract SARS-CoV-2 nucleocapsid proteins from the specimen by mixing it with the extraction buffer provided with the product. However, the remnant NP specimen after RT-PCR was not enough; therefore, the volume of the extraction buffer added was 200 μL for MARK-B™ COVID-19 Ag, and the sample was 1:1 diluted after UTM is added. For the Standard Q COVID-19 Ag Test, the volume of extraction buffer is 200 μL and 200 μL of UTM is use, thus the dilution factor is 1:1 [https://www.who.int/diagnostics_laboratory/eual/eul_0563_117_00_standard_q_covid19_ag_ifu.pdf].

Due to the dilution, the direct swab protocol usually offers better results for most of the commercial antigen tests [https://www.medrxiv.org/content/10.1101/2021.05.12.21257107v1.full#ref-5]. According to the clinical study done by Samkwang Medical Lab (South Korea), for the nasopharyngeal swab specimens from the United States, the direct swab protocol for MARK-B™ COVID-19 Ag provided high sensitivity and specificity (94.4% sensitivity and 100.0% specificity). In addition, when the MARK-B™ COVID-19 Ag is operated by minimally trained operators at the CLIA-waived environments (Hometown Urgent Care, OH), the sensitivity and the specificity were 91.4% and 100.0%, respectively. However, the we believe that the direct comparison of the results obtained in this study with the previous research is not relevant, as the specimens used in these studies were not identical.

We described this in revised manuscript (line 99-103, 121-125)

3. Ct should be proportional to Log2(RNA concentration) since it represents the number of RNA doubling steps required to achieve a detectable PCR signal. It is therefore probably most appropriate to plot Log2(MESIA current) vs. Ct, which should provide a slope approaching 1. If the log(current) vs. Ct is not linear, that should be discussed.

**Answer :** We thank the reviewer for the comment**.** We plotted log2(current) values vs. Ct values and fitted with a linear line. While the signal was saturated at the concentrations higher than Ct 20, the log2(current) vs. Ct showed a linear relationship in the range from Ct 20 to Ct 33, as shown below:

We described this in revised manuscript (line 194, 261-263)

4. In the methods section, it isn't very clear how the MESIA technique works. I think readers would provide a little more detail or, alternatively, a reference to where they could go for more details.

**Answer :** In MESIA (Magnetic force-assisted electrochemical sandwich immunoassay), when a sample is loaded into a cartridge, it flows into the microchannel containing gold-coated magnetic nanoparticles and an electrochemical sensor. Target analytes are actively mixed with the magnetic nanoparticles via external magnetic fields. They form immuno-complexes on the electrochemical sensor through antibody-antigen interactions. Finally, the target analytes are quantified by measuring the electrochemical redox signal from the gold-coated magnetic nanoparticles bound to the sensor electrode. All the processes from sample preparation to analysis are automatically conducted in a hand-held reader system, the MARK-B™ 1 analyzer.

We described this in revised manuscript (line 84-86, 90-93)

Previous literatures regarding MESIA:
<https://pubmed.ncbi.nlm.nih.gov/30926043/>
<https://www.clin-lab-publications.com/article/3100>
